# Incompleteness

**Published:** 1888-03-24

**Authors:** 


### INCOMPLETENESS.

NOT he who first beholds the aloe grow,
May think to gaze upon its perfect flower;
He tends, he hopes, but e'er the blossom blow
There needs a century of sun and shower.

He shall not see the product of his toil,
Yet were his work neglected, or ill-done,
Did he not prune the boughs and dig the soil,
That perfect blossom ne'er might meet the sun.

Perhaps he has no prescience of its hue,
Nought of its form and fragrance may foretell;
Yet, in each sun-shaft, in each bead of dew,
Faith, passing knowledge, tells him he does well.

Our lives, O fellow-men, pass even so.
We watch and toil, and for no seeming gain;
The future, which no mortal can foreknow,
May prove our labour was not all in vain.

But what we sow we may not hope to reap,
Perfect fruition may not seek to win,
Not till, work-weary, we have fallen asleep,
Shall blossom blow, or fruit be gathered in.

Let it be so! Upon our darkened eyes
A light more clear than noon-tide rays shall shine,
If pain of ours have helped our race to rise,
By just one hair's-breadth, nearer the divine.

Upward and outward, plant-like, life extends,
Grows fairer as it doth the more aspire;
Never completed, ever-more it sends
A branch out, striving higher still and higher.

Because so great it must be incomplete,
Have endless possibilities of growth;
Strength to grow stronger, sweetness still more sweet,
Yearning towards God, Who is the source of both.

